# Comparison of toxic responses to acetaminophen challenge in ICR mice originating from different sources

**DOI:** 10.1186/s42826-019-0017-x

**Published:** 2019-09-03

**Authors:** Tae Bin Jeong, Joung-Hee Kim, Sou Hyun Kim, Seunghyun Lee, Seung Won Son, Yong Lim, Joon-Yong Cho, Dae Youn Hwang, Kil Soo Kim, Jae-Hwan Kwak, Young-Suk Jung

**Affiliations:** 1 College of Pharmacy, Pusan National University, Busan, 46241 South Korea; 2 Department of Clinical Laboratory Science, College of Nursing and Healthcare Science, Dong-Eui University, Busan, South Korea; 3 Exercise Biochemistry Laboratory, Korea National Sport University, Seoul, South Korea; 4 Department of Biomaterials Science, College of Natural Resources & Life Science/Life and Industry Convergence Research Institute, Pusan National University, Miryang, South Korea; 5 College of Veterinary Medicine, Kyungpook National University, Daegu, South Korea; 6 College of Pharmacy, Brain Busan 21 Plus Program, Kyungsung University, Busan, South Korea

**Keywords:** Acetaminophen, Hepatotoxicity, GSH, ICR mouse

## Abstract

Acetaminophen (APAP) is the most common antipyretic analgesic worldwide. However, APAP overdose causes severe liver injury, especially centrilobular necrosis, in humans and experimental animals. At therapeutic dosage, APAP is mainly metabolized by sulfation and glucuronidation, and partly by cytochrome P450–mediated oxidation. However, APAP overdose results in production of excess reactive metabolite, N-acetyl-*p*-benzoquinone imine (NAPQI), by cytochromes P450; NAPQI overwhelms the level of glutathione (GSH), which could otherwise detoxify it. NAPQI binds covalently to proteins, leading to cell death. A number of studies aimed at the prevention and treatment of APAP-induced toxicity are underway. Rats are more resistant than mice to APAP hepatotoxicity, and thus mouse models are mainly used. In the present study, we compared the toxic responses induced by APAP overdose in the liver of ICR mice obtained from three different sources and evaluated the usability of the Korl:ICR stock established by the National Institute of Food and Drug Safety Evaluation in Korea. Administration of APAP (300 mg/kg) by intraperitoneal injection into male ICR mice enhanced CYP2E1 protein expression and depleted hepatic GSH level 2 h after treatment accompanied with significantly increased level of hepatic malondialdehyde, a product of lipid peroxidation. Regardless of the source of the mice, hepatotoxicity, as evidenced by activity of serum alanine aminotransferase, increased from 8 h and peaked at 24 h after APAP treatment. In summary, hepatotoxicity was induced after the onset of oxidative stress by overdose of APAP, and the response was the same over time among mice of different origins.

## Introduction

Acetaminophen (APAP) is the most popular analgesic and antipyretic. Generally, the recommended dose is 4 g/day for adults and 60–75 mg/kg/day for children. However, overuse of APAP can cause acute liver failure followed by hepatocellular necrosis [1–4]. Clinically, the most common cause of APAP addiction are suicide attempts (47.8%) in adolescents and accidental addiction (42.2%) in infants [5]. The only medication in cases of APAP overdose is N-acetyl cysteine, a well-known antioxidant. Thus, the development of new drugs superior to NAC is urgently required. Our PubMed search using the MeSH terms ‘liver injury’, ‘hepatotoxicity’, and ‘acetaminophen’, suggested that intensive research has been undertaken to understand the mechanism of APAP-induced acute hepatic injury and develop novel drug candidates.

The cause of hepatotoxicity induced by over-dosage of APAP is the excessive generation of toxic substances mediated by xenobiotic metabolism, also known as drug metabolism, in the liver [6, 7]. At the suggested dosage, APAP is mainly metabolized by sulfation and glucuronidation, and partly by cytochrome P450–mediated oxidation. However, APAP overdose results in production of excess reactive metabolite, N-acetyl-*p*-benzoquinone imine (NAPQI), by cytochromes P450. NAPQI overwhelms the level of the endogenous tripeptide glutathione (GSH), which could otherwise detoxify it. NAPQI binds covalently to proteins, leading to cell death [8–11]. Interestingly, the toxic effects of acetaminophen differ in different mammalian species [12–16]. For example, mice are highly vulnerable to liver injury induced by APAP in comparison with rats [16]. Although the overall metabolism of APAP is similar in both species, mice exhibit severe mitochondrial dysfunction accompanied by high oxidative stress, similar to what occurs in humans [17]. Therefore, it is reasonable to use mice in preclinical studies of APAP-induced hepatotoxicity.

Outbred stocks of experimental animals are commonly used to study genetics, oncology, toxicology, and pharmacology in academy and industry. ICR mice, originated from albino non-inbred mice in Switzerland, are a vigorously outbred stock [18]. The National Institute of Food and Drug Safety Evaluation (NIFDS) in Korea has established the “Korl:ICR” stock and it was used for the last 50 years in the NIFDS. Recently, it has been proved that Korl:ICR mice are genetically independent from commercially available ICR stocks from Japan and USA; however, no biological differences have been observed so far [19]. The purpose of the present study was to characterize the Korl:ICR stock in terms of hepatotoxicity and determine its utility in evaluating the safety of preclinical drug candidates. Because APAP-induced hepatotoxicity is involves excessive oxidative stress following GSH depletion [20], we examined the level of GSH and lipid peroxidation in the liver as well as the parameters of hepatotoxicity including the activity of alanine aminotransferase (ALT) in serum and histopathological analysis.

## Materials and method

### Animals and treatments

Male ICR mice were obtained from three different sources. Korl:ICR was supplied by the Department of Laboratory and Animal Resources at the NIFDS (Cheongju, Korea). The other two groups of ICR stocks were purchased from several suppliers located in the United States (A: ICR) and Japan (B: ICR). The use of animals complied with established guidelines and was approved by the Institutional Animal Care and Use Committee of Pusan National University (PNU-2018-1992). The basic conditions such as facility environment and diet were as described previously [21]. Mice were acclimated to room temperature (22 ± 2 °C) and humidity (55 ± 5%) with a 12-h light–dark cycle for 1 week prior to use in the animal facility of the university. They were provided free access to water and standard irradiated chow diet (Samtako Inc., Osan, Korea) consisting of moisture (12.5%), protein (25.43%), fat (6.06%), fiber (3.9%), ash (5.31%), calcium (1.14%), and phosphorus (0.99%). Mice received a single intraperitoneal injection of APAP (300 mg/kg) or vehicle, and samples were collected at 2, 8, 24, and 48 h after APAP administration.

### Examination of APAP-induced hepatotoxicity

Blood samples were collected at 2, 8, and 24 h after injection and immediately transferred to BD Microtainer Blood Collection Tubes (BD Life Sciences, Franklin Lakes, NJ, USA). They were centrifuged at 3000×*g* for 15 min to separate the serum, and the activity of serum ALT was examined by the method of Reitman and Franke [22]. For histological evaluation, liver samples were fixed in 10% phosphate buffered formalin overnight and embedded in paraffin. The tissue was cut into 4–mm-thick sections and stained with hematoxylin and eosin: the nucleus was stained blue with Mayer’s hematoxylin, while the cytoplasm was stained red with eosin (Sigma-Aldrich). Histopathological analysis was performed using an inverted microscope (Olympus, Tokyo, Japan).

### Measurement of GSH in liver tissue

Total GSH was determined as reported previously [21]. The liver was homogenized with 4 volumes of ice-cold 1 M perchloric acid. The denatured protein was removed by centrifugation at 10,000×*g* for 10 min and the supernatant was analyzed for total GSH concentration using HPLC separation followed by fluorescence detection. After addition of 10 μL of 10% (w/v) tris (2-carboxyethyl) phosphine hydrochloride solution, the samples were incubated for 30 min. Next, 90 μL of trichloroacetic acid solution containing 1 mM EDTA was added, and the sample was centrifuged (13,000×*g*, 10 min). Fifty microliter of supernatant was then added to 10 μL of NaOH (1.55 M), 125 μL of 0.125 M borate buffer (pH 9.5) and 50 μL of 0.1% (w/v) 7-fluorobenzofurazan-4-sulfonic acid ammonium salt (0.125 M with 4 mM EDTA). Samples were incubated at 60 °C for 1 h and then injected into HPLC equipped with a fluorescence detector (excitation, 385 nm; emission, 515 nm) (FLD-3100; Thermo Scientific, Sunnyvale, CA, USA). Chromatographic separation was carried out using a Hector M-C18 column (3 μm × 4.6 mm × 150 mm) (Chiral Technology Korea, Daejeon, Korea).

### Measurement of malondialdehyde (MDA) in liver tissue

Hepatic level of MDA was determined as reported previously [23]. The liver was homogenized with a triple volume of 1.15% KCl. The dispensed homogenate was mixed with 0.2% thiobarbituric acid in 2 M sodium acetate buffer containing 5% butylated hydroxytoluene. The mixture was incubated at 95 °C for 45 min. The supernatant after centrifugation of mixture was injected into the HPLC equipped with a fluorescence detector and a 5 μm Symmetry C18 reverse phase column (4.6 mm × 150 mm, Eka Chemicals, Bohus, Sweden). The mobile phase consisted of 35% methanol and 65% sodium phosphate buffer. The MDA–thiobarbituric acid complex was monitored by fluorescence detection with excitation at 515 nm and emission at 553 nm.

### Western blotting

Western blotting was accomplished as reported previously [23]. Liver was lysed with ice-cold protein extract solution (iNtRON; Sungnam, Gyunggi, Korea), and the protein concentration was examined by the BCA reagent (Thermo Scientific, Sunnyvale, CA, USA). Equal amounts of protein were separated by SDS-PAGE and transferred onto a nitrocellulose membrane (Bio-Rad, Hercules, CA, USA). The membranes were incubated with TBS-T containing 5% milk and the primary antibodies against CYP2E1 (Detroit R&D, Detroit, MI, USA), GAPDH (Santa Cruz Biotechnology, Santa Cruz, CA, USA). After washing, the membrane was incubated with the horseradish peroxidase-conjugated secondary antibodies. The antigen was detected using a Western Bright ECL HRP substrate kit (Advansta, Menlo Park, CA, USA).

## Results

### APAP-induced hepatic injury in mice of different sources increased ALT

**Fig. 1:**
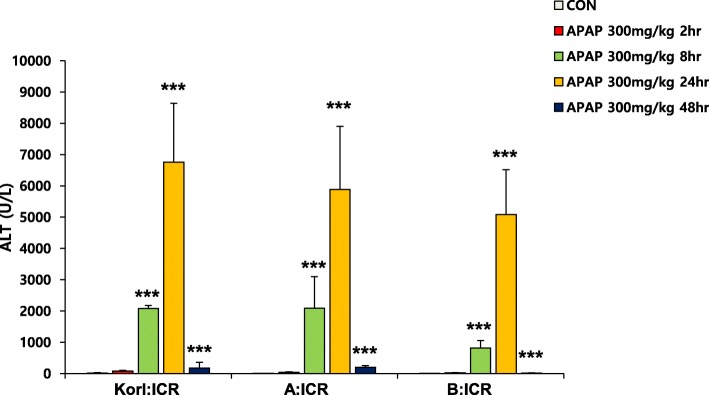
Effect of APAP on serum activity of ALT. Each value represents the mean ± standard deviation for six mice. *** Significantly different from the corresponding control (Student’s *t*-test, *p* < 0.001)

ALT, a biochemical parameter of hepatotoxicity, increased from 8 h after APAP injection and peaked at 24 h in all mice regardless of their source (Fig. 1). However, it returned to the normal level at 48 h after treatment.

### Histopathological changes in the liver of APAP-treated ICR mice

**Fig. 2:**
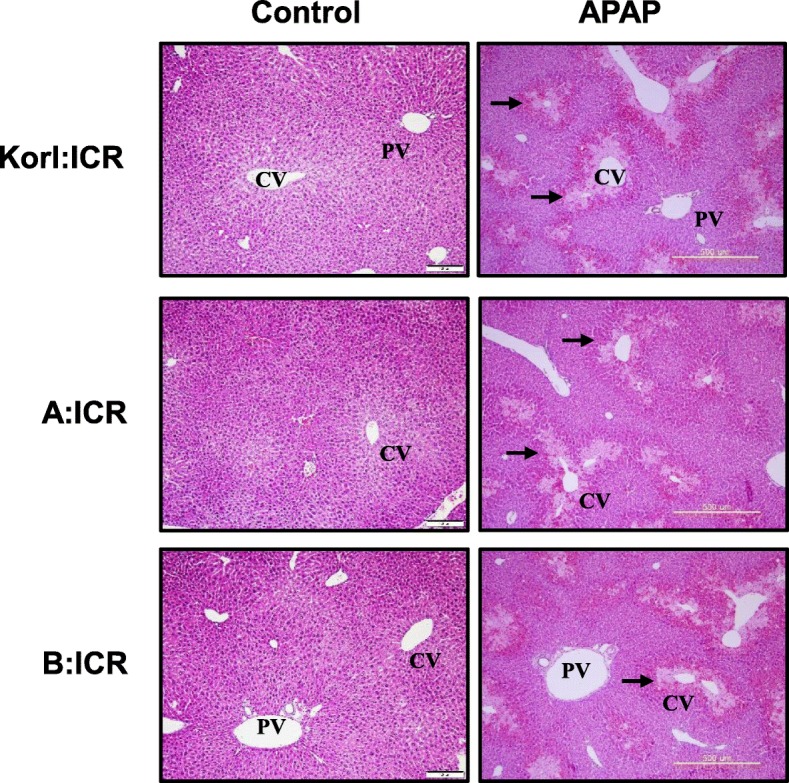
Histopathological changes in liver tissues at 24 h after APAP treatment. Liver sections from the control and APAP-treated Korl:ICR, A:ICR and B:ICR mice were stained with hematoxylin and eosin. CV, Central vein; PV, portal vein. Arrows indicate necrotic area. Original magnification, × 100

Based on the result of serum ALT activity, we accomplished histopathological analysis using liver tissues obtained from APAP-treated mice for 24 h and compared with vehicle-treated control mice. At 24 h after injection, the necrotic area of the central vein in the liver of APAP-treated mice was considerably larger than that in the liver of vehicle-injected mice (Fig. 2). There were no notable differences among the three sources of mice.

### Dynamics of hepatic CYP2E1 protein expression and GSH levels after APAP treatment

**Fig. 3:**
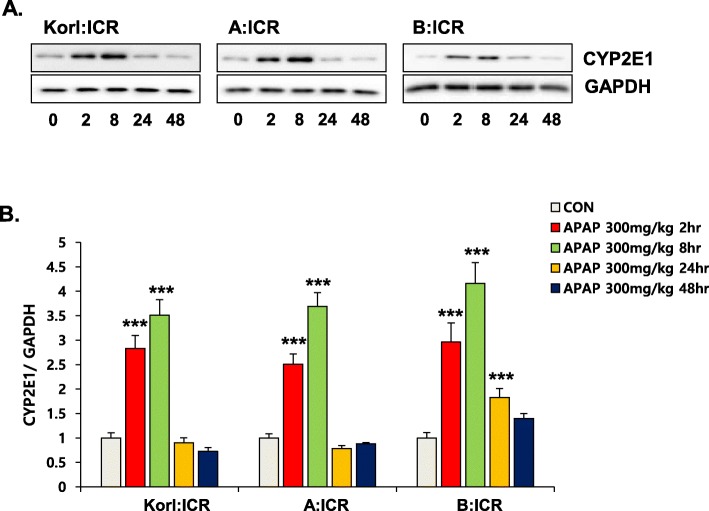
Effect of APAP on the expression levels of CYP2E1 protein in the liver. (**a**) Western blot analysis of CYP2E1 in the liver of APAP-treated mice for 48 h and (**b**) quantification of CYP2E1 levels. Quantitative analysis of blots normalized by GAPDH expression. Each value represents the mean ± standard deviation (SD) for six mice. *** Significantly different from the corresponding control (Student’s *t*-test, *p* < 0.001)

**Fig. 4:**
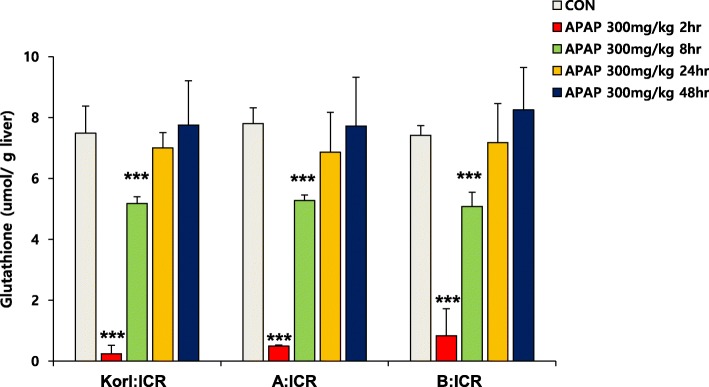
Effect of APAP on GSH levels in the liver. Each value represents the mean ± standard deviation (SD) for six mice. *** Significantly different from the corresponding control (Student’s *t*-test, *p* < 0.001)

Large doses of APAP is correlated with increased generation of a reactive metabolite, NAPQI, through enzyme reactions mediated by CYP enzymes [24]. Although various forms of CYP are implicated in the oxidative metabolism of APAP, it is generally accepted that CYP2E1 plays a principal role in production of NAPQI [25]. Subsequently, it is detoxified via conjugation with an endogenous tripeptide, GSH. After APAP injection, protein level of CYP2E1 increased from 2 h and it was highest at 8 h after treatment (Fig. 3). The hepatic GSH level rapidly decreased, reaching minimum at 2 h, but was restored at 8 h to control level (Fig. 4). Changes in hepatic GSH content induced by APAP treatment were similar in ICR mice from the three sources.

### Dynamics of hepatic MDA levels after APAP treatment

**Fig. 5:**
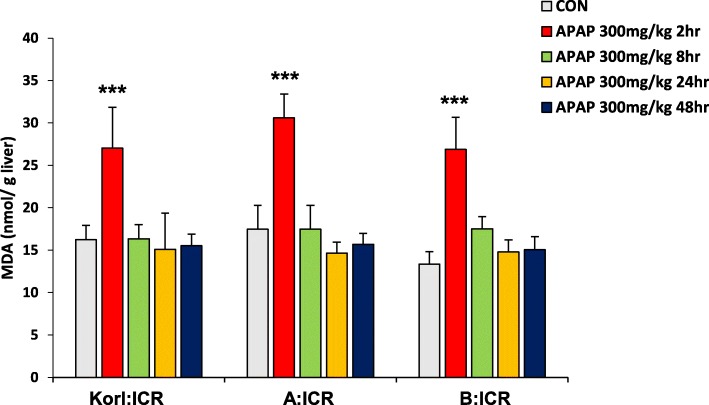
Effect of APAP on MDA levels in the liver. Each value represents the mean ± standard deviation (SD) for six mice. *** Significantly different from the corresponding control (Student’s *t*-test, *p* < 0.001)

The depletion of GSH by NAPQI is an important component of APAP-induced oxidative stress followed by liver injury. MDA level, an index of oxidative stress, was significantly increased at 2 h after APAP treatment (the time point of maximum hepatic GSH depletion), but returned to normal at 8 h (Fig. 5), confirming that MDA reflects the changes in antioxidant capacity of the liver. When it was compared among three different sources of ICR mice, No significant difference was observed among ICR mice from the three sources.

## Discussion

GSH is a tripeptide composed of cysteine, glutamic acid, and glycine and exists in all mammalian tissues; its levels are particularly high in the liver [26]. It is a major endogenous antioxidant that protects cells from oxidative stress or electrophilic metabolites from xenobiotics. GSH is synthesized by γ-glutamylcysteine ligase (GCL) and subsequently GSH synthetase as a final product in the transsulfuration pathway [27]. APAP is partly metabolized by cytochrome P450 (CYP) enzymes such as CYP1A2, CYP2E1, and isoforms of CYP3A, to the highly reactive metabolite NAPQI [28]. In normal physiological conditions, NAPQI is rapidly conjugated with GSH mediated by glutathione S-transferase. The resulting conjugate, APAP–GSH, can be further metabolized to APAP–cysteine/cysteinylglycine and APAP–mercapturate followed by excretion.

Overdosed APAP leads to increased bioactivation by CYP enzymes and formation of NAPQI, and also causes depletion of cellular GSH. Excess NAPQI may result in hepatotoxicity due to covalent binding to essential cellular macromolecules and mitochondrial dysfunction, and/or other mechanisms such as oxidative stress [27, 29]. In particular, recent studies have focused on the effect of NAPQI on mitochondria, which produce ATP through respiration [16, 17, 30, 31]. NAPQI caused by APAP overdose depletes mitochondrial GSH, and remaining NAPQI binds to mitochondrial proteins including the membrane ones. Subsequently, increased reactive oxygen species generated by GSH-depleted mitochondria activate JNK and induce translocation to mitochondria, where JNK induces permeabilization of the mitochondrial membranes and inhibits mitochondrial bioenergetics, eventually leading to cell death due to depletion of ATP [16, 17, 30, 31].

Drug-induced liver injury (DILI) is a major clinical and regulatory issue worldwide [32]. Control of DILI is still not easy because of the poor understanding of the mechanisms of toxicity caused by different drugs. Without knowledge of these mechanisms, it is difficult to develop ways to predict and prevent problems. To understand the causes of DILI, a research model that can reproduce the clinical response is indispensable. APAP is the most commonly used model drug of dose-dependent and predictable DILI. Indeed, the mechanism of APAP toxicity has been well studied, but the clinical problem has not been solved completely and APAP overdose is the primary cause of acute liver failure in several countries [33]. Early studies that investigated APAP-induced hepatotoxicity used multiple species, including rats, guinea pigs, cats, and hamsters, but currently it is clear that mice are the best available model [17] .

In the present study, we compared the time course of hepatic injury following oxidative stress induction by APAP in ICR mice from three different sources. Serum activity of ALT and histopathological analysis were used to determine liver injury, and hepatic levels of GSH and MDA were analyzed to examine the involvement of oxidative stress. Increased oxidative stress, suggested by increased CYP2E1 expression, hepatic GSH depletion and MDA elevation at 2 h after APAP administration, preceded liver injury, which was observed from 8 h, and hepatotoxicity, which peaked at 24 h. This study confirms that Korl:ICR mice are comparable to ICR mice from the two other sources in terms of APAP-induced hepatotoxicity.

## Conclusions

The results of this study clearly show that APAP overdose induces hepatic GSH depletion accompanied by oxidative stress and followed by liver injury. We also found that the responses of Korl:ICR mice established by the NIFDS in Korea are similar to those of ICR mice from other sources, which suggests that it is a useful resource to study the mechanism of DILI and chemical-induced hepatotoxicity.

## Data Availability

The datasets used and/or analyzed in this study are available from the corresponding author on reasonable request.

## Abbreviations

ALT: Alanine aminotransferase
APAP: Acetaminophen;
CYP: Cytochrome P450
DILI: Drug-induced liver injury
GCL: γ-glutamylcysteine ligase
GSH: Glutathione
MDA: Malondialdehyde
NAPQI: N-acetyl-*p*-benzoquinone imine
NIFDS: National Institute of Food and Drug Safety Evaluation
